# Prevalent neuropathy in a cohort of HIV-infected Kenyan sex workers using antiretroviral drugs

**DOI:** 10.11604/pamj.2016.25.14.9699

**Published:** 2016-09-20

**Authors:** Frank Ndaks Ndakala, Julius Otieno Oyugi, Margaret Ng’wono Oluka, Joshua Kimani, Alexandra Jablonka, Georg Martin Norbert Behrens

**Affiliations:** 1 University of Nairobi, Institute of Tropical and Infectious Diseases, Directorate of Research Management & Development, State Department of Science & Technology, Nairobi, Kenya; 2 University of Nairobi, Institute of Tropical and Infectious Diseases, University of Manitoba, College of Medicine, Department of Medical Microbiology, Winnipeg, Manitoba, Canada; 3 University of Nairobi, College of Health Sciences, School of Pharmacy, Nairobi, Kenya; 4 University of Manitoba, College of Medicine, Department of Medical Microbiology, Winnipeg, Manitoba, Canada; 5 Clinical Immunology and Rheumatology, Hannover Medical School, Germany and German Centre for Infection Research, Germany; 6 Clinical Immunology and Rheumatology, Hannover Medical School, Germany and German Centre for Infection Research, Germany

**Keywords:** Antiretroviral therapy, height, risk factor, resource-limited settings, sex workers, polyneuropathy, stavudine, tenofovir disoproxil fumarate

## Abstract

**Introduction:**

Several risk factors including stavudine and age have been strongly associated with polyneuropathy. However, conflicting data exist on height as an independent risk factor in polyneuropathy. The objective of this study is to exclude height as an independent polyneuropathy risk factor in a cohort of human immunodeficiency virus (HIV)-infected Kenyan sex workers.

**Methods:**

This was an analysis of prospectively collected data of treatment-naive subjects initiating either stavudine or tenofovir diphosphate fumarate or zidovudine-based antiretroviral therapy (ART) regimens from January 2008 to August 2012. Polyneuropathy was characterised as burning sensation, numbness, or dysesthesia. The study used arithmetic means of weight (kg) and height (cm) measured in duplicates using calibrated scales.

**Results:**

After exclusion of duplicate data sets and un-confirmed cases of polyneuropathy, the study identified 212 patients without polyneuropathy, 14 pre-ART and 94 post-ART related polyneuropathy cases. Polyneuropathy cases were older but did not differ in demographic, clinical and laboratory parameters at baseline. There was a significant difference in first-line ART regimens with more patients on tenofovir disoproxil fumarate in the post-ART group (p=0.017).

**Conclusion:**

Polyneuropathy is a common disorder among HIV-infected Kenyan sex workers. These data cannot support the postulated increased risk by height after matching for gender and ART duration. Though stavudine is associated with polyneuropathy, in this study many patients previously not exposed to stavudine developed polyneuropathy. This suggests the involvement of unknown risk factors such as genetic and metabolite differences in the development of polyneuropathy.

## Introduction

Global access to antiretroviral therapy (ART) has dramatically lowered the mortality and morbidity rates of human immunodeficiency virus (HIV)-infected patients [1]. However, with substantially expanding life expectancy particularly in resource-limited settings including Kenya, the burden of polyneuropathy (PN) can complicate the effectiveness of many treatment programs. Sensory neuropathies including PN are the most frequent neurological disorders associated with HIV infection and its treatment [2]. Because of the severe pain associated this condition, PN severely affects the quality of life and daily function of people living with HIV-infection [3].

Two major types of HIV-associated PN exist: primary HIV-associated [3] or toxic types associated to nucleoside reverse transcriptase inhibitors (NRTIs), particularly the “D-drugs” including zalcitabine (ddC), stavudine (d4T), and didanosine (ddI). Both types of PN affect approximately 30-67% of HIV-infected patients [4, 5]. There are no estimates of the burden of HIV-related PN for the Kenyan sex workers whose HIV prevalence is approximately 29.3% [6]. In addition, scarce data exist on risk factors associated with PN among this population. PN is the most frequent ART-related toxicity in sub-Saharan Africa [7, 8], especially in older patients [9].

Exposure to d4T is a well-established independent risk factor for PN among many cohorts in both resource-rich [10, 11] and resource-limited settings [8]. However, not all patients receiving d4T end up with PN, suggesting that host factors may play a role in the patient’s risk. Widely studied examples of host factors include the presence of mitochondrial haplogroup T [12], genetic markers of host inflammatory responses and cytokine genotypes, notably alleles of tumor necrosis factor-A (TNFA) [13–15]. In the general population, several factors including diabetes mellitus, poor glycemic control, male sex, white race, and older age can increase the risk of developing PN [16, 17]. In the HIV-infected population, more advanced HIV disease or AIDS, CD4 cell count<100 cells/mm^3^, viral load above 10, 000 copies/ml, past history of neuropathy, use of other neurotoxic drugs e.g. anti-tuberculosis drugs, certain nutritional deficiencies (vitamin B-12 deficiency), co-existing conditions such as diabetes or hepatitis C and alcoholism have been associated with PN [18].

There is adequate literature on some PN-related risk factors such as d4T use, CD4 cell counts, and older age. However, conflicting data exist on how PN is associated with height in the aging HIV-infected population. Whereas several studies have consistently associated height with increased PN risk [19–21], other studies have consistently found no association between height and PN [22]. The diagnosis of PN did not require the presence of symptoms in studies where height was not associated with PN [9]. Height has proved to be an important and practical predictor of other forms of neuropathy [17, 22, 23]. There are suggestions that it increase the risk of neuropathy because of increased axon surface exposure to toxins [24]. In a study that confirmed height as a risk factor, there was a significant association between height and PN despite their cohort being 5 cm shorter on average than in other ethnic groups [20]. The authors explain this as an influence of longer leg length relative to the trunk length in Black compared with White individuals [25].

The purpose of the current study was to exclude height as an independent risk factor in a cohort of HIV-infected Kenyan sex workers, after matching them for gender and duration of therapy and after excluding other risk factors such as alcohol or diabetes.

## Methods

### Study design

This was a retrospective study that utilized routinely collected data from HIV-infected sex workers at Pumwani clinic were analysed. Pumwani clinic is part of the sex workers outreach program (SWOP) managed by both the University of Manitoba from Canada and University of Nairobi, Institute of Tropical and Infectious Diseases (UNITID) from Kenya. We selected Pumwani clinic because of its well-established cohort with a large number of patients on ART [26]. A clinician examined all ART-eligible patients prior to initiating first-line ART that based on public sector standards and clinician’s judgment in line with national guidelines and drug availability. The extraction of patient data was from the Kenya AIDS Control Project database.

### Eligibility criteria

Patients were eligible for this study if they were HIV-infected, ART-naive, ≥18 years of age and initiated on a standard first-line regimen of stavudine (d4T) or zidovudine (AZT) or tenofovir disoproxil fumarate (TDF) with lamivudine (3TC) and either efavirenz (EFV) or nevirapine (NVP) between January 2008 and August 2012. The analysis excluded patients with suspected or confirmed active tuberculosis, disorders of the central nervous system, current, or history of diabetes, viral hepatitis, liver cirrhosis, vitamin B-12 deficiency, renal failure, cancer, hypothyroidism and history for alcohol intake.

From a sex worker population of 6 202 individuals in the Pumwani cohort, those who developed PN from January 2008 to August 2012 were identified by clinicians from the database and diagnosis of PN confirmed during the next routine visit. The study considered the date of ART initiation as a baseline and used same population to select patients without PN (controls) by matching cases for gender and time on ART. A ratio of two controls for every case was projected. Extraction of data including demographic, laboratory and clinical characteristics occurred from participants’ case report forms and for up to seven follow-up visits with a mean interval of about 5 to 6 months.

### Anthropometric measurements

Using calibrated scales, weighing of patients was free of any heavy items and after removing shoes. Measurements of current weight (kg) and height (cm) were in duplicate based on procedures recommended by WHO [27]. We used the arithmetic means obtained from the two measurements then calculated body mass index (BMI) by dividing the weight in kg by the square of the height in metres.

### Assessment of neuropathy

The diagnosis of PN relied on clinicians’ judgment and at least one of the following lower limb neuropathic clinical symptoms: numbness, dysesthesia, burning sensation, stabbing pain or aching, pins and needles. Clinicians in SWOP clinics have had intensive training in the history, clinical presentation, examination, and treatment of PN. We classified subjects as pre-ART PN if records showed a clinical diagnosis of PN before ART initiation. The Post-ART PN group included patients whose records showed a clinical diagnosis of PN after initiation of ART.

### Ethics approval

The Kenyatta National Hospital / University of Nairobi-Ethics Research Committee approved the study protocol and all study subjects gave written informed consent on confidential use of information extracted from their medical records.

### Data analysis

Data were analysed using SPSS software, version 20 (IBM, SPSS. New York, USA). Frequencies, percentages for categorical variables, measures of central tendency and measures of variability for continuous variables, were analysed. Statistical analysis for pairs of continuous variables and non-parametric variables relied on student’s t-test (or Mann-Whitney for non-normally distributed data) and *χ*² tests respectively.

## Results

After exclusion of patients with complete lack of laboratory data during the ART initiation, patients with duplicate data sets, and un-confirmed symptoms for polyneuropathy (PN), 320 patients (n=212 without PN, n=14 pre-ART PN, n=94 post-ART PN) were identified (Table 1).

**Table 1:** Baseline characteristics of HIV-infected Kenyan patients on Long-Term ART

Baseline characteristics [Mean (SD)]	No PN (n=212)	PN-before ART (n=14)	PN-After ART (n=94)	Total N=320	Differences No PN vs. PN after ART
Age (years)	36.8±7.9	40.0±10.2	39.2±8.0	37.6±8.2	**t=-2.40 p=.017**
Gender (Female) [n(%)]	148 (69.8%)	14(100%)	68(72.3%)	230(71.9%)	χ=0.2 p=0.685
Height (cm)	163.0±8.8	159.6±11.8	162.1±8.1	162.6±8.7	T=.83 p=.405
BMI (kg/m^2^)	24.1±4.3	24.8±3.5	24.4±4.4	24.3±4.3	T=-.51, p=.608
CD4 count (cells/mm^3^)	240.0±102.4 (n=125)	279.3±123.5 (n=7)	235.4±103.4 (n=64)	239.9±103.2 (n=196)	T=.30 p=.766
Hemoglobin (g/dL)	12.7±2.7 (n=125)	13.3±3.2 (n=7)	12.4±2.4 (n=64)	12.6±2.6 (n=196)	T=.61 p=.583
White blood cell count (tsd. cells/µl)	5.4±2.5 (n=125)	5.5±1.7 (n=7)	5.6±2.6 (n=64)	5.5±2.5 (n=196)	T=-.55 p=.583
Platelet count (tsd. cells/µl)	294.4±100.2 (n=118)	339.6±87.2 (n=7)	280.9±101.4 (n=57)	291.9±100.2 (n=182)	T=.83 p=.407
CD4/CD8 ratio	0.28±0.17 (n=97)	0.26±0.13 (n=7)	0.29±0.18 (n=53)	0.3±0.2 (n=157)	T=-.28 p=.778
ART-initiated					
AZT-Based	91(42.9%)	4(28.6%)	34(36.2%)	129(40.3%)	χ=1.23 p=0.314
d4T-Based	104(49.1%)	8(57.1%)	43(45.7%)	155(48.4%)	χ=0.29 p=0.593
TDF-Based	17(8%)	2(14.3%)	17(18.1%)	36(11.3%)	**χ=6.68 p=0.017**
3TC-Based	212(100%)	14(100%)	94(100%)	320(100%)	
NVP-Based	182(85.8%)	12(85.7%)	76(80.9%)	270(84.4%)	χ=1.23 p=0.307
EFV-Based	30(14.2%)	2(14.3%)	18(19.1%)	50(15.6%)	χ=1.23 p=0.307
Time to first change of ART regime (months)	37.7±13.8	32.4±18.5	34.7±17.0	36.8±14.9	T=1.67 p=.097
ART-duration (months)	47.6±10.4	48.2±6.9	48.4±10.0	47.9±10.2	T=-.64 p=.525

SD-standard deviation; n-number; BMI-body mass index; cm-centimeter; kg/m^2^-kilogram per square meter; bpm-beats per minute; mmHg-millimeters of mercury; CD4-cluster of differentiation-4; CD8-cluster of differentiation-8; g/dL-grams per deciliter; tsd cells/µl-thousand cells per microliter; AZT-zidovudine; d4T- stavudine; 3TC-lamivudine; TDF-tenoforvir disoproxil fumerate; NVP-nevirapine; EFV-efavirenz; ART-antiretroviral therapy; PN-polyneuropathy

Patients who developed PN during treatment did not differ at baseline in terms of gender, height, body mass index (BMI), CD4 count, ha emoglobin, white blood cell count, platelet count, CD4/CD8 ratio, initial ART (except TDF), time to first change of ART regime and ART duration overall from patients without PN over the course of the study. Patients with PN were older than the ones that did not develop PN. There was a significant difference in first-line ART regimen, with more patients on TDF based ART in the PN after treatment group. The study found no difference in other components of the first-line ART regime (Table 1).

**Figure 1:**
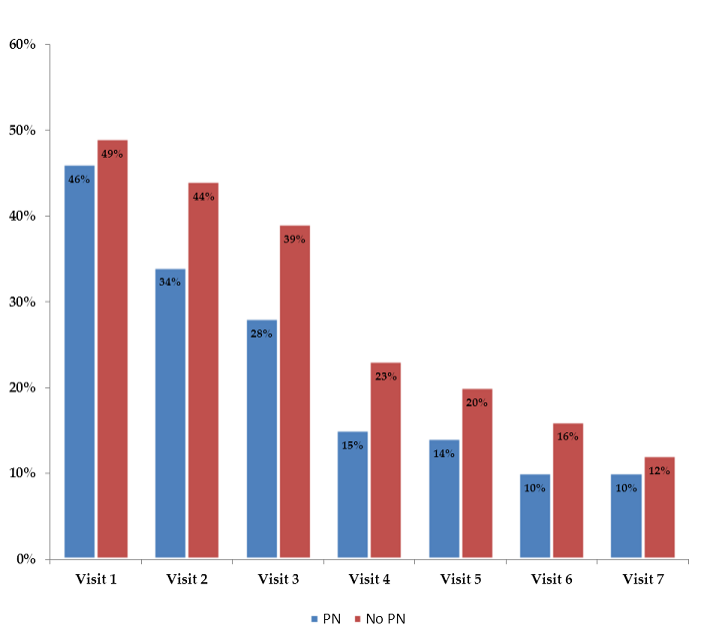
Percentage of stavudine-containing regimens during seven visits by HIV-infected Kenyan patients (PN-polyneuropathy)

In the gender and ART duration matched patients, with the exclusion of other known factors of PN height was not significantly different in patients who developed PN. At baseline, 48.4% of all patients were on d4T. There was no significant difference between patients who received d4T and the controls. Patients who developed PN rapidly and more frequently switched for d4T compared to patients who did not develop PN (Figure 1). The main factor for change of d4T was the change of ART policy in line with World Health Organization’s recommendations of 2010 [28].

**Figure 2:**
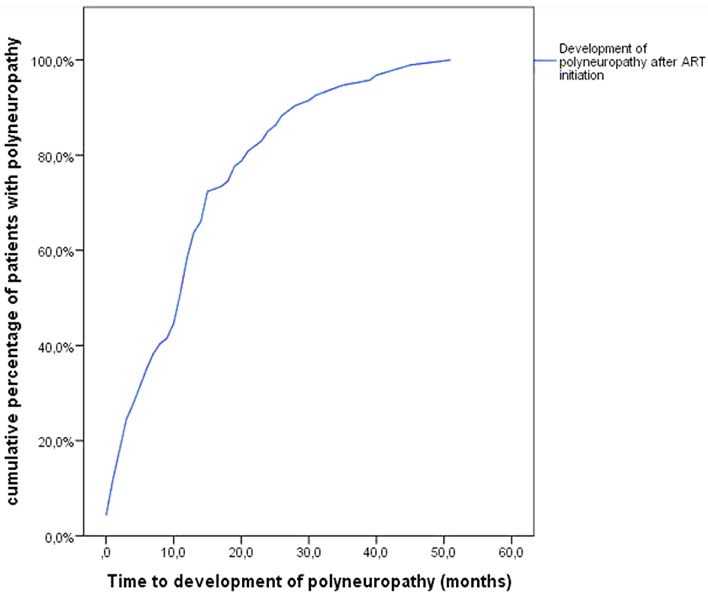
Time to development of polyneuropathy in HIV-infected Kenyan patients

The mean time to development of PN was 13.1(±11.4) months after initiation of ART (Figure 2). The mean follow-up time was 4.0 years in all groups.

## Discussion

The current retrospective cohort study that aimed at excluding height as an independent risk factor of PN found out that PN was a common disorder in HIV-infected Kenyan sex workers. These findings confirmed that PN is independently associated with increasing age but not height as postulated. The study also reports a significant difference in first-line ART regimen with more patients on TDF in the post-ART group. Although more patients were using d4T (48.4%) at baseline, there was no significant difference between patients who received d4T and controls. Although d4T is strongly associated with PN, these findings failed to confirm this association. Further, many patients previously not exposed to d4T ended up developing PN. Therefore, PN will remain a burden even with the rapid scale-up of ART that has so far transformed HIV-infection from a terminal illness to a chronic disease [29].

PN is a disabling neurological complication known to impair the quality of life of HIV-infected populations [30, 31]. It is widely under-detected, undocumented and poorly treated in most resource-limited settings. Studies conducted in similar settings have shown variations in the prevalence of PN. Some studies have reported PN prevalence rates of between 2 % and 42% [20, 32–37]. PN prevalence rates ranging from 35-52% recorded in Western countries among ART-naive patients, were similar to those reported in developing countries [3, 33]. Such variations in prevalence rates are likely to result from a combination of factors including variations in the diagnosis of PN, different risk factors, and small sample sizes.

In the current analysis, age at baseline was significantly different in patients who developed PN versus controls. These findings support many previous studies that identified age as a risk factor for developing PN in HIV [2, 5, 20, 36, 37]. According to some authors, long, large and metabolically stressed peripheral nerves are likely particularly vulnerable to toxicity and damage caused by D-drugs [2]. So far, age stands out as the most notable and consistent risk factor for PN. With increased global access to ART and the rapidly aging HIV population on successful therapy, the association of aging as a risk factor in PN portends an ongoing burden from this complication for HIV therapeutics [2].

These results do not confirm the clinical impression that PN in HIV is length-dependent. However, a number of recent studies have associated patient height and risk of developing PN [2, 19–23]. According to these authors, taller patients are vulnerable to PN when exposed to d4T. The pathogenesis of the relation between height and PN remains conflicting. Some authors relate increased nerve length with greater axon surface area. They have suggested that any localized injury to an axon may impair the overall conduction properties of the nerve [24]. Therefore, patients with longer nerves (and thus a larger total axon surface area) may be at an increased risk of PN when exposed d4T [24]. In addition, greater leg length might relate to a prolonged time required to complete regeneration of any injured nerves. Alternatively, increased height could also relate to increased hydrostatic pressure experienced in the feet of tall patients when they stand up [38].

In almost all populations, height is significantly associated with gender, as the average female is smaller than the average male. However, even when gender is controlled, height remains a controversial independent risk factor. Unlike studies that found height as an independent risk factor in HIV-PN, several other studies reported no association. For instance, two Rwandese studies found no association between height and PN [39, 40]. Studies where gender was not controlled have produced controversial findings too. Mehta et al. showed that the incidence of PN during the first year of ART was 10 times higher in woman than in men [22]. Since increased height is associated with male patients, one would expect a high incidence of PN in male than female patients.

Exposure to “D drugs” including d4T has proved to be the most consistent risk factor associated with PN [7, 9, 41, 42]. However, this study did not find a similar association. Other studies that reported no association between d4T and PN include studies by Luma et al., [31] and Tumusiime et al., [39]. Lack of association was suggested to be due to the limited proportion of d4T users or other risk factors other than d4T [35, 43]. Further, the current findings suggest that the contradictory results observed in the above-mentioned studies, may be due to a confounder that is associated with height and PN, such as alterations in metabolites or genetic variance.

The present study excluded known risk factors for PN other than HIV-associated PN and matched patients for gender and ART duration. After adjusting for those factors, the postulated association of height and the development of PN remained undetected. More than 50% of the patients who developed PN in this cohort never had any d4T exposure. Moreover, d4T exposed patients largely did not develop PN. Even after excluding known risk factors for PN, a number of patients still developed PN. These findings support other studies that have reported the ongoing high prevalence of PN in the absence of d4T exposure [30, 33, 44–46]. For example, in a diverse cohort of more than 1 500 HIV-infected patients, more than half of the patients had PN [30]. Compared to earlier studies [33, 44–46], the prevalence of PN was not lower in this cohort even in the absence of d4T [30]. Before combination ART (cART), risk factors of PN included advancing age, immunosuppression, ongoing D-drug use, and elevated plasma HIV-viral load [47, 48]. These findings concurs with Ellis et al., that ongoing “D-drug” use is no longer associated with increased risk of PN [30]. Current CART use and previous D-drug exposure constitute new risk factors [30]. Moreover, the current findings reveal that even without exposure to d4T, some patients still develop PN. This suggests that unknown risk factors such as metabolites [43] or genetic variations may play a role in the development of PN. Considering that d4T is no longer first line treatment, it means that PN is still an ongoing burden for HIV therapeutics. Thus, there is a need for novel treatment modalities to promote regeneration of damaged neurons and manage neuropathic pain.

Inadequate PN case ascertainment and a modest sample size were limitations in the study. The use of clinical signs and symptoms in diagnosing PN is a considered best practice in HIV treatment and care. However, the diagnosis of PN was restricted on symptoms only. Most PN signs occur in the absence of symptoms. Therefore, by relying solely on the report of symptoms consistent with neuropathy the study may have ended up with a wrong estimation of overall cases of PN. Symptoms are not pathognomonic for PN, and consequently have low specificity and sensitivity [49, 50]. That explains why studies that used tests such as nerve conduction with laboratory evaluations for vitamin B-12 and diabetes had a higher prevalence of PN.

Additional limitations in the study included the inability to determine a causal effect relationship due to the retrospective nature of the study. Under- and over-detection of PN can be due to incomplete clinical records and biases in the clinicians’ judgment during matching. Taking into consideration these limitations, it could still show that when matching for gender and ART duration and excluding known risk factors, the proposed influence of height no longer influences the development of PN.

## Conclusion

HIV-associated polyneuropathy is a common disorder among HIV-infected Kenyan sex workers in Kenya. Data from the current study failed to support the postulated increased risk by height when matching for gender and ART duration. Although d4T can lead to toxic PN, many patients in the study who had no previous exposure to d4T developed PN. This suggests the involvement of unknown risk factors such as changes in metabolites or genetic factors in the development of PN. Therefore, there is a need for further research on the role of genetic variation and metabolite changes as risk factors of PN in HIV-infected populations.

### What is known about this topic

- Polyneuropathy affects HIV-infected patients before and after exposure to ART;
- The use of d-drugs including stavudine is strongly associated to post-ART related polyneuropathy;
- While pre-ART related polyneuropathy abates after initiation of ART, post-ART related polyneuropathy abates upon the withdrawal of the offending drug.

### What this study adds

- Polyneuropathy is a more common disorder in resource-poor settings (sex workers outreach programme health facilities);
- Some patients still develop polyneuropathy even when they are not exposed to offending drugs including stavudine;
- Older age is increasingly becoming an important a risk factor in aging patients on long-term ART.
